# Cardiovascular Safety and Effectiveness of Bisphosphonates: From Intervention Trials to Real-Life Data

**DOI:** 10.3390/nu14122369

**Published:** 2022-06-07

**Authors:** Chiara Delli Poggi, Maria Fusaro, Maria Cristina Mereu, Maria Luisa Brandi, Luisella Cianferotti

**Affiliations:** 1 Bone Metabolic Diseases Unit, Department of Experimental, Clinical and Biomedical Sciences, University of Florence, 50139 Florence, Italy; chiara.dellipoggi@unifi.it; 2 National Research Council (CNR), Institute of Clinical Physiology (IFC), 56124 Pisa, Italy; dante.lucia11@gmail.com; 3 Department of Medicine, University of Padova, 35128 Padova, Italy; 4 Independent Researcher, 09100 Cagliari, Italy; crissmer5412@gmail.com; 5 F.I.R.M.O. Italian Foundation for the Research on Bone Diseases, 50141 Florence, Italy; marialuisa@marialuisabrandi.it

**Keywords:** osteoporosis, fragility fractures, zoledronate, alendronate, risedronate, cardiovascular diseases

## Abstract

Both osteoporosis with related fragility fractures and cardiovascular diseases are rapidly outspreading worldwide. Since they are often coexistent in elderly patients and may be related to possible common pathogenetic mechanisms, the possible reciprocal effects of drugs employed to treat these diseases have to be considered in clinical practice. Bisphosphonates, the agents most largely employed to decrease bone fragility, have been shown to be overall safe with respect to cardiovascular diseases and even capable of reducing cardiovascular morbidity in some settings, as mainly shown by real life studies. No randomized controlled trials with cardiovascular outcomes as primary endpoints are available. While contradictory results have emerged about a possible BSP-mediated reduction of overall mortality, it is undeniable that these drugs can be employed safely in patients with high fracture risk, since no increased mortality has ever been demonstrated. Although partial reassurance has emerged from meta-analysis assessing the risk of cardiac arrhythmias during bisphosphonates treatment, caution is warranted in administering this class of drugs to patients at risk for atrial fibrillation, possibly preferring other antiresorptives or anabolics, according to osteoporosis guidelines. This paper focuses on the complex relationship between bisphosphonates use and cardiovascular disease and possible co-management issues.

## 1. Epidemiology of Fragility Fractures and Cardiovascular Diseases: Introduction and Brief Overview

Osteoporosis is characterized by a reduction in bone mineral density, deterioration of bone microarchitecture, and an increased risk of fragility fractures (FF), which mostly involve spine, hip, distal forearm, and proximal humerus, globally indicated as major osteoporotic fractures. FF lead to acute pain, loss of function, disability, and hospitalization, being recognized as a major cause of morbidity and mortality [1].

Worldwide, osteoporosis causes over 9 million fractures a year. In 2015, 15.8 million women and 4.2 million men had osteoporosis in Europe; the number of estimated FF in the same area in 2017 was 2.7 million, equivalent to 7332 fractures/day. Almost the 66% of these fractures occurred in women, with hip and vertebral ones accounting for 19.6% and 15.5% of all fractures, respectively [2].

The incidence of FF is rising in many countries, likely due to the longevity of the population in the last decades, since osteoporosis involves, above all, the elderly population. According to World Health Organization (WHO) data, the proportion of the population aged more than 60 years, mostly targeted by FF, will double from 12% in 2015 to 22% in 2050, namely from the current 900 million to 2 billion (https://www.who.int/news-room/fact-sheets/detail/ageing-and-health, accessed on 15 February 2022).

Consequently, the number of total FF will rise of 23.3% in the next years (from 2.7 million in 2017 to 3.3 million in 2030). In particular, the increase is 28% for hip fractures and 23% for spine fractures. This is reflected by an increase in fracture-related costs, which in Europe amounted to €37.5 billion in 2017 and are projected to increase to €47.4 billion in 2030 (+27%) [2,3].

For all these reasons, osteoporosis and FF burden is widely recognized as a serious public health issue.

In parallel, chronic non-communicable diseases, which include cardiovascular diseases (CVD), are rapidly rising in all ages and particularly in elders. As a matter of fact, osteoporosis and CVDs often coexist in elderly population.

Recent data from the American Heart Association show that the prevalence of CVD (comprising coronary heart disease, heart failure, stroke and hypertension) in adults is 49.2% overall and increases with age [4]. Interestingly, it has been estimated that the lifetime risk of hip fracture at age 50 years was comparable to the lifetime risk of stroke for both women (20%) and men (14%) in the European area and, generally, the lifetime risk of major osteoporotic fracture was comparable to that of CVD—29% for women and 38% for men [5].

Traditionally, osteoporosis and CVD have been considered separated entities within non-communicable diseases, linked only by the increase in prevalence in relation to old age. Despite this, several common risk factors and pathophysiological aspects and possible common pathogenetic mechanisms have been suggested in the last few years [6].

The aim of this paper will be to review the state-of-the-art on the relationship between cardiovascular morbidity and mortality after bone fragility events and the possible modulation of cardiovascular issues by bisphosphonates (BSPs), the most commonly drugs for osteoporosis.

## 2. Cardiovascular Morbidity and Mortality after Fragility Fractures

Osteoporotic FF are often associated with increased morbidity [7]. With respect to CVD, many studies have shown that the risk of cardiovascular events (CVE) is increased after FF, in particular after a hip fracture. 

A retrospective case-control study in Taiwan showed that hip fracture is independently associated with a 29% increase in risk of developing acute myocardial infarction compared with controls [8]. Another prospective case-control study showed that patients with hip fracture have a higher risk (1.55 times) of stroke in the year after the event [9]. These data suggest that the imminent risk of CVE parallel the risk of refracture after a major osteoporotic fracture [10,11].

The mechanisms for the association between osteoporotic FF and CVDs are still unknown, and more studies are necessary to clarify the pathogenetic relationship, especially in osteoporotic post-menopausal women, who seem to have higher risk of CVD than osteoporotic men. Some data suggest that estrogen deficiency is a key factor for atherosclerosis progression and development of bone fragility since estrogen receptors are expressed both in osteoclasts/osteoblasts and in endothelial and smooth muscle cells of blood vessels with key roles in the modulation of the function of these cells [12].

FF, especially hip ones, are associated with increased mortality rates, which increases from 30 days to some years after the fracture according to different studies. An Australian case-control study conducted on an elderly and institutionalized population showed that the risk of death remained high for 9 months after a hip fracture, with the main causes of death being infections for females and, most of all, CVDs for both males and females [10].

Another cohort study in England has investigated the cause of death for up to one year after the first fracture (hip, wrist, humerus, spine, ribs, or pelvis). One-year mortality risk following fracture increased with age and was more than threefold higher as compared to the general population, with the major causes of death being neoplasms, respiratory diseases, and CVDs, similarly both in men and women [11].

On the other side, to strengthen the link between these two conditions, relevant data suggest a major risk of FF in patients with CVDs. In a cross-sectional study, after adjusting for age and cardiovascular risk factors, high coronary artery calcium score and obstructive coronary artery disease, revealed by computed tomography, have found to be associated with low bone mineral density in asymptomatic post-menopausal women without previous FF and CVDs [13]. Similarly, women with CVD have been shown to have increased risk of FF [14]. Recently, a relationship between iliac arteries calcifications and abdominal aortic calcifications on one side and the risk for vertebral fractures on the other has been demonstrated in hemodialysis patients, even more so after adjusting for vitamin K, a major player both in bone and vascular health [15].

Currently, there are no recommendations for screening and treatment of cardiovascular risk factors and diseases in osteoporotic patients and vice versa, but we should consider these aspects in clinical practice, in order to mitigate morbidity and mortality of the osteoporotic population.

## 3. Bisphosphonates in the Prevention of Fragility Fractures

**Figure 1:**
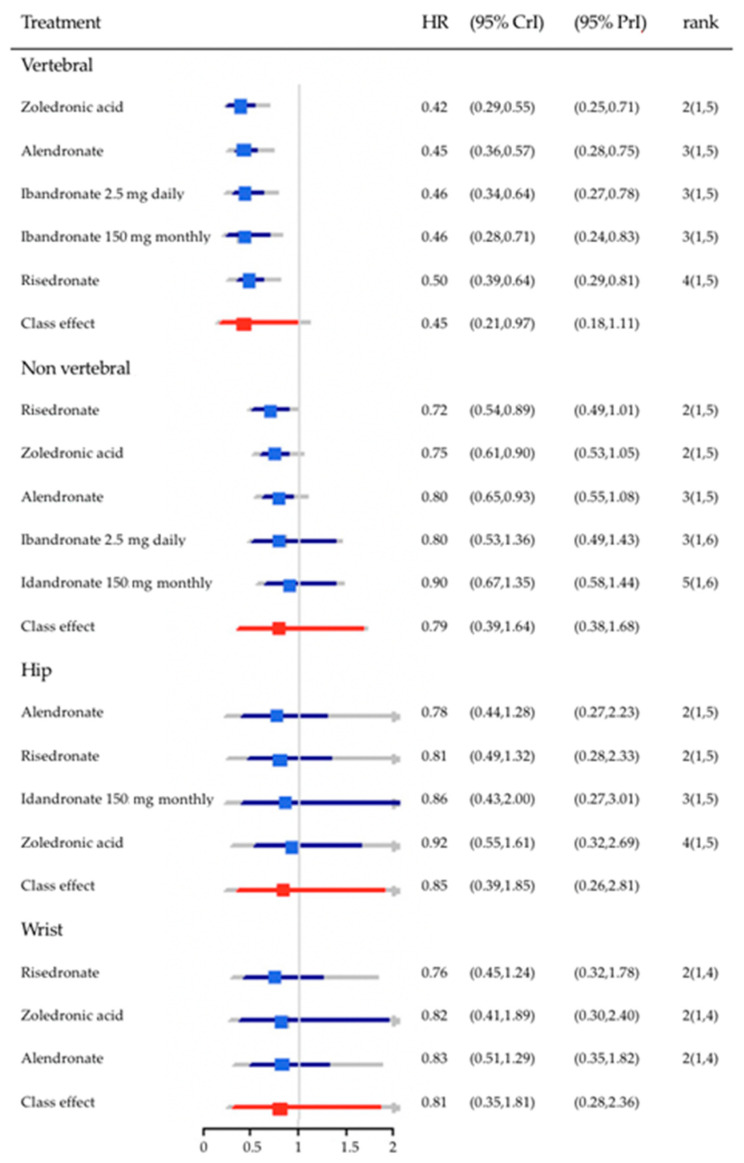
Hazard ratios (HR) and 95% credible intervals (CrI) for the effect of treatment relative to placebo by fracture outcomes. Blue—pooled effects; Red—class effects; Grey: 95% prediction intervals (PrI); median ranks and 95% CrI are displayed in the right hand column (as reprinted with permission from Ref. [17]. Copyright 2022, Elsevier).

BSPs are the most widely used agents for treatment of osteoporosis in Europe and worldwide. They are stable analogues of pyrophosphate, characterized by a P-C-P bond. They are potent inhibitors of bone resorption, by reducing recruitment and activity of osteoclasts and inducing their apoptosis. The relative potency of bone resorption inhibition and bone affinity are higher in nitrogen-containing BSPs (NCBSPs), or amino-BSPs, such as alendronate, risedronate, ibandronate, and zoledronic acid [16] (Figure 1, as reprinted with permission from Ref. [17]. Copyright 2022, Elsevier).

The oral BSPs alendronate 70 mg once weekly and risedronate 35 mg once weekly (75 mg twice monthly) are the most common used BSPs worldwide [18].

Alendronate has been shown to reduce the incidence of vertebral and non-vertebral fractures by half in women with prevalent vertebral fractures, accordingly to Fracture Intervention (FIT) study [19,20]. In a case-control analysis alendronate also reduced the risk for hip fractures by 34% in the oldest old patients [21].

Risedronate reduces the incidence of vertebral and non-vertebral fractures by 40–50% and 30–36%, respectively, in women with previous prevalent vertebral fractures and decreases significantly the risk of hip fractures by 30%, especially in osteoporotic women between 70 and 79 years of age [22,23,24].

Oral ibandronate (administered 150 mg monthly) reduces the risk of vertebral fractures by 50–60% and was first approved only for the prevention of vertebral fracture in postmenopausal osteoporosis; however, a post-hoc analysis of long-term data (5 years) concluded that time-to-fracture was significantly longer for all clinical fractures versus placebo [25,26].

Clodronate is a weak bisphosphonate (BSP) that decreases the risk of vertebral and non-vertebral fractures in randomized controlled studies, but it is licensed for use in osteoporosis only in few countries [27].

Finally, zoledronic acid, given in intravenous infusion once a year (5 mg), has shown to reduce the incidence of vertebral fractures by 70% and the incidence of hip fractures by 40% in postmenopausal osteoporotic women (HORIZON Pivotal Fracture Trial) [28]; moreover, it reduces the risk of fracture when given shortly after a first hip fracture in both sexes (HORIZON Recurrent Fracture Trial) [29].

The recent guidelines for the diagnosis and management of postmenopausal osteoporosis, published in 2019, recommend establishing osteoporosis treatment based on the risk of fracture over a ten-year interval (low, high, or very high risk), calculated using FRAX algorithm and bone mineral density (BMD). People with a prior FF should be treated without further assessment [18]. Unfortunately, despite these recommendations and the clear link between prior FF and subsequent osteoporotic fractures, as well as the association with increased morbidity and mortality, only a few patients with non-traumatic fractures undergo further evaluation and adequate treatment for osteoporosis. According to the literature, only just over 20% of patients with fragility fracture receives a proper anti-osteoporosis medication, and many studies of the last few years demonstrate that this percentage is almost stable, showing that little progress has been made in improving management of osteoporotic FF [30]. Moreover, adherence to antiosteoporosis medications is low, especially when orally administered [31].

## 4. Bisphosphonates and Mortality

Beyond the reduction of FF, some studies suggest that BSPs also reduce mortality. 

The first evidence of a mortality reduction in people with FF receiving treatment with BSPs derived from the HORIZON Recurrent Fracture Trial [29]. In this study, patients with a recent hip fracture, treated with zoledronic acid once yearly within 90 days after the event, were shown to have a reduction of 28% in deaths from any cause in addition to the 35% risk reduction of any new clinical fracture in a median follow-up of 1.9 years. The percentage of deaths from cardiovascular and cerebrovascular events in the zoledronic acid group was slightly lower than in the placebo group, even if not statistically significant [29]. Data from literature are discordant and, apart from the HORIZON Recurrent Fracture Trial, no other randomized controlled trials (RCTs) of BSPs detected the same effect on mortality. Nonetheless, the results from the HORIZON Recurrent Fracture Trial sparked a meta-analysis, which, including 8 RCTs, published up to 2008 with antiresorptives (risedronate, zoledronate, strontium ranelate, and denosumab) found that antiresorptive treatments reduce mortality by 11% in frail, older individuals at increased risk for fracture, independently of age or incident fractures [32]. When the analysis was restricted to BSPs, the mortality benefit appeared similar, even because data from studies of non-BSP antiresorptives were fewer.

Osteoporotic FF are known to increase mortality, thereby reducing the risk of new clinical fractures by means of antiosteoporosis drugs could also lead to a lowering in number of deaths. Currently, it is unclear whether this effect is a consequence of abatement of new fractures’ number or a direct result of pharmacological intervention with BSPs.

Interestingly, a recent post-hoc analysis of data from an RCT assessing the effect of zoledronic acid administered intravenously every 18 months in osteopenic women showed a significant reduction in mortality rate in the subset of patients without incident FF [33].

A conspicuous number of real-life, observational studies shows that treatment with BSPs is associated with a reduction in mortality. 

Brozek and colleagues found out that mortality was significantly lower in patients with hip fracture treated with any type of BSP, whether it started before or after the event [34]; similar data were previous obtained by Beaupre and colleagues describing mortality reduction in patients with hip fracture exposed to oral BSPs [35] and by Sambrook and colleagues describing a 27% reduction in risk of death in institutionalized older people using BSPs compared with non-users [36].

Goodbrand and colleagues demonstrated that BSP use was significantly associated with time to death and reduced mortality among older people undergoing rehabilitation [37], while in a previous study, death resulted at a significantly reduced rate only in women treated with BSPs after a fracture, but not in men [38]. Moreover, Lee and colleagues found a lower in-hospital mortality in critically ill patients that used pre-admission BSPs when compared with non-users [39]. More recently, Bergman and colleagues have shown that BSP use was associated with lower mortality in hip fracture patients discharged from hospital, but only within days of treatment initiation, although the association was not significant within weeks [40].

Despite these real-life results, a recent meta-analysis by Cummings and colleagues, including 27 clinical trials and 56,737 participants, concluded that no significant association subsists between BSPs and overall mortality, suggesting that BSPs cannot be recommended to increase life-span but only to reduce fracture risk [41].

In conclusion, even if some previous studies have reported that BSPs are associated with reduced mortality in addition to reduced fracture risk, currently there are no sufficient data to recommend the treatment for this reason alone, regardless of the personal risk of fracture. Conversely, it can be stated that mortality rates are no higher in BSP users, reassuring healthcare providers on their use in clinical practice to reduce fracture risk.

## 5. Bisphosphonates and Cardiovascular Disease

**Figure 2:**
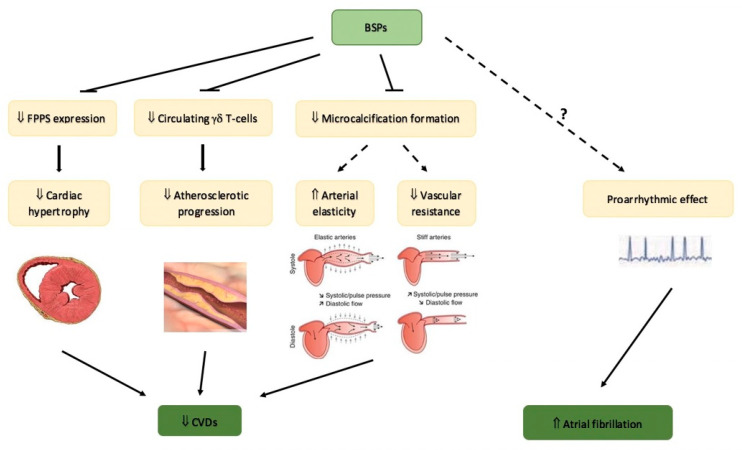
Potential mechanisms involved in the relationship between bisphosphonates (BSPs), cardiovascular diseases (CVDs), and arrhythmias, according to data from preclinical studies, are shown. BSPs have shown to reduce cardiac hypertrophy, atherosclerotic plaque, and vascular resistance and to improve arterial elasticity; potential mechanisms of the proarrhythmic effect of BSPs are not yet clear. FPPS = farnesyl pyrophosphate synthase. Dotted arrows indicate uncertain mechanism and relationship, and arrows next to the words show the increase or decrease. “?” indicates uncertain mechanisms and effects.

The relationship between BSPs and CVD is related to the potential atherosclerotic protection by BSPs on one side and the possible association with increased risk of atrial fibrillation (AF) on the other [42]. In Figure 2, possible mechanistic connections between the use of BSPs and CVD are shown.

### 5.1. Bisphosphonates and Cardiovascular Protection

Given the possible relationship between CVD and the use of BSPs, preclinical studies have tried to examine in vivo in animals the effects of these drugs. A number of studies have consistently shown that administering BSPs to vertebrates (pigeons, monkeys, etc.) fed an atherogenic diet lead to a reduction of atherosclerotic plaque size and percentage, improvement of arterial elasticity, decrease in systemic vascular resistance and carotid-artery intima-media thickness and overall decrease in intravascular calcifications (as reviewed in [42]). As far as the possible pathogenetic mechanisms are concerned, while overexpression of farnesyl pyrophosphate synthase (FPPS) results in cardiac hypertrophy in mice, the targeted inhibition of FPPS by NCBSPs results in an attenuation of cardiac hypertrophy through inhibition of the mevalonate pathway leading to prevention of ischemia-induced myocardial remodeling [43]. Moreover, a decrease in circulating γδ T-cells, which are known to stimulate atherosclerotic progression, has been demonstrated under BSP treatment [44]. Recently, an in vitro model of vascular calcification, employing three-dimensional collagen hydrogels incubated with calcifying extracellular vesicles, BP (ibandronate) treatment significantly modulated in a time-dependent way microcalcification formation. The final effect was dependent on the initiation of BP treatment, i.e., inhibition or enhancement of microcalcifications before or after the microcalcification formation itself, respectively [45]. Nonetheless, these studies have generally employed higher doses of BSPs with respect to the ones administered in humans to decrease fracture risk, so that it is not possible to directly infer and explain possible benefits of BSPs on cardiovascular system [42]. Herein, evidence coming from intervention trials in osteoporosis and real-life data have to be considered in order to assess the possible link between the use of BSPs and CVD. 

Cardiovascular outcomes have not been established as primary outcomes in pivotal clinical trials of BSPs nor with other anti-osteoporosis drugs. Post-hoc analyses have mainly focused on the safety of BSPs when cardiovascular outcomes were considered. Indeed, in the HORIZON Pivotal Fracture Trial, the use of zoledronate administered intravenously once-yearly did not affect progression of abdominal aortic calcification as assessed by spine lateral X-rays, originally performed to assess incident vertebral fractures, over the 3-year study period [46].

Since cardiovascular calcifications correlate with atherosclerosis disease burden, studies have assessed whether the use of BSPs could be associated to increased prevalence of vascular calcification, with contradictory results. A meta-analysis including 61 trials reporting the effects of BSPs on atherosclerotic process found that BSPs may reduce arterial wall calcifications but have no effect on arterial stiffness [47]. In 2010, Elmariah et al. [48] demonstrated that in 3710 ethnically diverse women belonging to the Multi-Ethnic Study of Atherosclerosis (MESA) study group, the use of NCBSPs was associated with decreased and increased prevalence of vascular calcifications in older and younger women, respectively, suggesting an age-dependent relationship between the use of these drugs and atherosclerosis. Examining these effects in a cohort of kidney transplant recipients randomized to BSP treatment or placebo for secondary osteoporosis with high fracture risk, the progression of abdominal aortic calcification, a known predictor of cardiovascular mortality, was almost completely inhibited over the 24-month study period [49].

In a retrospective cohort study of a large public healthcare database based in Hong Kong, the risk of CVD was assessed in a population of patients with hip fracture under alendronate (*n* = 4594) versus untreated patients (*n* = 13,568). Alendronate was shown to be associated with a significantly lower cardiovascular mortality and incident myocardial infarction at 1 year, which persisted in the long-term although at a lower level, with slightly lower reduction in the risk of stroke at 5 and 10 years after hip fracture [50]. These results were confirmed and consistent also when all patients under all NCBSPs were globally analyzed. Thus, administering NCBSPs after a hip fracture might decrease cardiovascular risk, which rapidly rises in these patients after the fracture occurrence. In this sense, the possible protection by alendronate from CVE might jeopardize the interpretation of the outcomes of recent post-hoc analyses, demonstrating an increased cardiovascular risk in patients treated with romosozumab, a novel anti-osteoporosis medication, when compared to alendronate alone in the assessment of the relationship between this novel treatment and potential cardiovascular adverse events [51].

In a recent analysis of result of the prospective cohort Odense Bisphosphonates Safety Study (OBSS), integrating data from the Danish national prescription registry enriched with local hospital data, hospitalizations for any CVE as primary outcome and specific CVE as secondary outcomes in a population of individuals older than 45 years undergoing DXA testing were taken into account. A 33% reduction in the risk of CVE in the 10-year study period was observed in BP’s users as compared with matched controls, further underlying the possible cardioprotective effects of this class of drugs [52]. 

A cohort study was performed on 82,704 subjects older than 4 years, incident users of BSPs, with cumulative doses, as indirect measures of drug adherence, expressed as proportion of days covered (PDC), as retrieved from an Italian regional healthcare database, with a mean follow-up over 6 years. The association between cumulative time-dependent exposure to BSPs and number of hospitalizations for atherosclerotic CVE was estimated by a multivariate Cox model. A significant decrease in the risk of hospitalizations was observed for individuals with intermediate (40–80%) and high (>80%) PDC (HR (hazard ratio) of cardiovascular (CV) hospitalization of 0.95 (0.91–0.99) and 0.75 (0.71–0.81), respectively), independently of age and sex. In the group with higher PDC, a reduced incidence of both cerebrovascular and coronary events was demonstrated. The authors concluded that high adherence to BDPs treatment was linked to a better cardiovascular outcome [53].

As far as adherence to BSPs is concerned, another result comes from another nested case-control study (multicenter I-GRaDE project) assessing the effect of the administration of BSPS in patients with pre-existing cerebrovascular disease. This study was carried out in a cohort of patients older than 65 years discharged from hospitals for cerebrovascular events in a 3-year period and assessed for subsequent hospital admissions for similar events. Interestingly, although the risk for cerebrovascular events was overall slightly higher in BPS users (identified as the ones with at least one prescription of these drugs), when adherence to BSPs was taken into consideration, a decreased risk of cerebrovascular events was observed in BSP users with a PDC above 80% (OR (odds ratio) = 0.81, of 0.95 (0.71–0.92)) [54]. Adherence bias could possibly influence the outcomes of the different studies and explain some of the contradictory results in this field. Indeed, another study focusing on the risk of stroke in BSP users belonging to the large electronic dataset collected by primary care physicians in England (*n* = 31,414). In patients older than 18 years with at least a stroke event in a 4-year interval, considered as cases, BSP treatment in the medium-term was not associated with increased risk of stroke, as compared to matched controls [55]. Previous studies demonstrated a lower risk of stroke and myocardial infarction, respectively, in patients on BSP therapy for prior osteoporotic fractures as compared with matched controls (untreated for vertebral or hip fractures) during a 2-year follow-up period, underlying that pre-existing baseline conditions such as fractures, linked to imminent/very high refracture risk, might also influence the observed results [9,56]. With this respect, BSPs can offer a protection against CVE in patients with baseline increased CV risk, as shown in patients with previous fractures [9].

Further RCT are necessary in this field to confirm whether adherence to BSPs is linked to decreased cardiovascular risk in the medium-long term and to explain the differences observed for different BSPs with different adherence rate. 

The fact that adherence to BSPs has not been systematically considered in most studies might explain conflicting results that have been shown when assessing safety and potential benefits of BSPs on CVD incidence and outcomes.

Remarkably, when zoledronate, an NCBSP with high adherence rate, is administered (5 mg i.v. at a 18 months interval), consistent results have been shown in a recent RCT carried out in women with osteopenia, as assessed by DXA (T-score between −1.0 and −2.5), aged 65 years or more [57]. In this study, in addition to a decreased number of recurrent fractures, fewer CVEs were observed in women receiving zoledronate versus the ones receiving placebo, together with a decreased incidence of cancer and an overall slight reduction in mortality rate, as further underlined in a subsequent in-depth analysis [57]. Specifically, the risk of myocardial infarction was higher in the placebo group than in zoledronic acid group (HR 0.60 (95% CI (confidence interval), 0.36–1.00)). When a composite cardiovascular endpoint was considered (myocardial infarction, coronary artery revascularization, sudden death, or stroke) 69 women suffered 98 events in the placebo group, and 53 women suffered 71 events in the zoledronate group (HR 0.76 (95% CI, 0.53 to 1.08)) with a decreased HR for death in the treated group of 0.65 versus 0.51 in the untreated (95% CI, 0.40 to 1.06; *p* = 0.08), and 0.51 (95% CI, 0.30 to 0.87) in the subset of patients without incident FF [33].

These novel and promising results encourage to possibly perform intervention trials with BSPs with high adherence with cardiovascular outcomes as primary endpoints, but, in the meantime, reinforce the attitude to appropriately treat patients with high fracture risk, even because of the possible non-skeletal benefits.

### 5.2. Safety of Bisphosphonates on Cardiovascular System: Atrial Fibrillation

The major concern in the short-term for treatment with BSPs, in particular with NCBSPs, is the possible association with arrhythmias, namely AF. In clinical practice, this represents a problem in the management of elderly patients with high fracture risk, in whom episodes of cardiac arrhythmias may have occurred. 

This concern was raised after post-hoc analyses of the RCT, demonstrating the efficacy of once-a-year zoledronate in the treatment of postmenopausal osteoporosis [28]. In this study employing zoledronate for primary prevention of FF (HORIZON Pivotal Fracture Trial), an increased risk of arrhythmia and, in particular, of AF was demonstrated in women receiving zoledronate versus women receiving placebo (6.9% versus 5.3%, respectively), with no correlation in time to exposure to the drug [28]. Hence, data from Fracture Intervention Trial were re-analyzed, confirming an higher incidence of AF in the alendronate-treated women versus controls [58]. An increased risk of AF was not observed in the re-assessment of trials employing risedronate as well as in the RCT testing zoledronate in patients of both sexes having suffered a recent hip fracture (HORIZON Recurrent Fracture Trial) [29]. Since these first trials, which led to indicate previous AF as contraindication for BSP administration, data with conflicting results have been published on this subject. One of these studies addressing this issue ascribed the higher occurrence of AF in patients after fracture because of a baseline increased risk of CVE in this category of patients [59]. Interestingly, in this study, the risk for AF was inversely correlated with adherence to BSPs [59]. Following this study, a meta-analysis re-assessing all the RCTs employing alendronate found no increased risk of AF for this drug in any single trial nor in the pooled analysis [60]. In the meta-analysis by Kim et al. [61], including 58 RCTs longer than 6 months in duration with all types of BSPs and assessing the safety of BSPs towards CVE and arrhythmias, these drugs were shown to be neutral as far as atherosclerotic events were considered, while a modestly elevated risk for AF was observed in zoledronate-treated patients. This might be in accordance with a recent retrospective cohort study taking advantage of a Danish healthcare database, which has found an increased rate of heart failure in zoledronate-treated patients, although a possible selection bias (i.e., higher cardiovascular risk at baseline in zoledronate users) cannot be excluded [62].

Nonetheless, in the majority of population-based case-control studies in real life, no evidence of increased risk of AF and flutter has been found overall in BPS users [63]. A recent systematic review has clearly highlighted the contradictory findings ensuing from RCT and mainly retrospective, real-life studies, as highlighted in Table 1 [64].

In conclusion, data from RCT and observational studies have failed to robustly show a strong, convincing association between BSP use and AF, as well as complete safety regarding this issue [42]. Nonetheless, in the presence of equivocal or conflicting data, BSPs’ prescription must be discouraged when a history of single-episode or recurrent AF is reported [78]. 

## 6. Conclusions

Both CVDs and bone fragility constitute hallmarks of advancing age. They can be associated in the same patients and can share mechanistic associations. It is therefore of a particular importance to clarify the possible effects and the safety of therapies employed in osteoporosis on atherosclerosis, CVD, and incident CVE.

BSPs, the larger group of effective antiresorptive drugs employed worldwide to decrease the risk of fracture, have been shown to be overall safe in the short-, medium-, and long-term and even decrease CVE and cardiovascular-related mortality in some settings, as demonstrated by post-hoc analyses of clinical trials and real-life studies. Adherence to BSPs, which is naturally higher for intravenous BSPs (i.e., zoledronate), and patient baseline clinical features, might be related to better CV outcomes. RCTs with CV outcomes as primary endpoints are both justified and necessary in this field [79].

Contradictory results have emerged from studies assessing the risk of cardiac arrhythmias, namely AF, still discouraging the administration of BSPs in patients at high risk for AF, as the ones having suffered previous similar events. 

As summarized in the key points (Table 2), far from recommending BSPs with the primary purpose of improving non-skeletal outcomes, the possible CV benefits and the possible effect on survival, ensuing from not-yet-defined extra skeletal mechanisms, together with the undeniable effectiveness in reducing risk for FF and refractures, make osteoporosis treatment a greater priority to be addressed in our healthcare systems. The benefits with the use of BSPs in patients with bone fragility greatly outweigh the possible risks in a usually compromised category of patients, with greater comorbidity and mortality.

## Figures and Tables

**Table 1:** Summary of clinical epidemiological studies investigating the relationship between BPs and AF (as reprinted with permission from Ref. [64]. Copyright 2022, John Wiley and Sons).

	Type of Study	Study Population	Suggested Relationship of BP in Causing AF
Black et al. [28]	Double-blind, placebo controlled	Osteoporotic patients	Proarrhythmic
Abrahamsen et al. [59]	Retrospective	Fracture patients	Nonarrhythmic
Sorensen et al. [63]	Retrospective	Atrial fibrillation/flutter patients	Nonarrhythmic
Bunch et al. [65]	Prospective	Coronary angiography patients	Nonarrhythmic
Grosso et al. [66]	Retrospective	BP patients	Nonarrhythmic
Wilkinson et al. [67]	Retrospective	Cancer patients	Proarrhythmic
Vestergaard et al. [68]	Retrospective	Osteoporotic patients	Nonarrhythmic
Huang et al. [69]	Retrospective	Osteoporotic patients	Nonarrhythmic
Erichsen et al. [70]	Retrospective	Cancer patients	Proarrhythmic
Lu et al. [71]	Retrospective	Osteoporotic patients	Proarrhythmic ^a^
Pazianas et al. [72]	Retrospective	BP users	Nonarrhythmic
Arslan et al. [73]	Cross-sectional	Cancer patients	Nonarrhythmic
Rhee et al. [74]	Retrospective	Osteoporotic patients	Antiarrhythmic
Herrera et al. [75]	Retrospective	Osteoporotic patients	Proarrhythmic
Wang et al. [76]	Retrospective	Osteoporotic patients	Proarrhythmic
Thadani et al. [77]	Prospective	Older male patients	Proarrhythmic ^b^

^a^ Lower dose was proarrhythmic, but higher dose was antiarrhythmic compared with raloxifene users. ^b^ Increase in nocturnal AF but no increase in clinically significant AF. BP, bisphosphonates; AF, atrial fibrillation.

**Table 2:** Relationship between cardiovascular outcomes and the use of bisphosphonates in clinical practice.

Bisphosphonates and Cardiovascular Outcomes
Observational data suggest that bisphosphonate (BSP) users may have lower mortality, delayed progression of vascular calcification, and atherosclerotic burden
Discrepancies exist between meta-analyses of RCTs and real-life studies on cardiovascular protection of bisphosphonates (due to different length of follow-up, different sampled populations, different treatment adherence, etc.)
There is not sufficient evidence to recommend antiresorptive treatment to reduce cardiovascular mortality (besides antifracture efficacy)
There is still not sufficient evidence to recommend antiresorptive treatment to reduce cardiovascular morbidity (besides antifracture efficacy)
No guidelines on osteoporosis focused on the cardiovascular safety of antiresorptives, especially in patients with concomitant CVDs or incident CVEs, nor address the issue on which antiresorptives can be administered as first choice (i.e., denosumab versus BSP)
Despite the fact that data on pro- or anti-arrhythmic effects of bisphosphonates are contradictory, it is advisable not to use these drugs in patients at high risk of AF

RCT, randomized controlled trial; CVD, cardiovascular diseases; CVE, cardiovascular events.

## Data Availability

Not applicable.
